# The Neuroprotective Effect of Tetramethylpyrazine Against Contusive Spinal Cord Injury by Activating PGC-1α in Rats

**DOI:** 10.1007/s11064-015-1606-1

**Published:** 2015-05-16

**Authors:** Jianzhong Hu, Ye Lang, Yong Cao, Tao Zhang, Hongbin Lu

**Affiliations:** Department of Spine Surgery, Xiangya Hospital, Central South University, Changsha, China; Department of Sports Medicine, Research Center of Sports Medicine, Xiangya Hospital, Central South University, No. 87 Xiangya Road, Changsha, 410008 China

**Keywords:** Tetramethylpyrazine, Contusive spinal cord injury, PGC-1α, Apoptosis, Neuronal survival

## Abstract

Tetramethylpyrazine (TMP) has been suggested to have neuroprotective effects against spinal cord injury (SCI); however, few studies have examined these effects and the corresponding mechanism. Therefore, the present study aimed to investigate the neuroprotective effect and underlying mechanism of TMP against contusive SCI. Adult male Sprague–Dawley rats were randomly divided into Sham, normal saline (NS) and TMP groups. Each group was divided into subgroups according to the time of sacrifice: 1, 3, 7, 14, 21 and 28 days post-injury. Laminectomy was performed in all groups, followed by contusive SCI establishment in the TMP and NS groups. TMP (80 mg/kg) was injected thereafter daily from 3 to 7 days post-injury in the TMP group, which was replaced by equal volume of normal saline in the NS group. The Basso–Beattie–Bresnahan (BBB) Locomotor Rating Scale was measured at different time points post-injury to appraise locomotor functional recovery. Quantitative real-time PCR and immunofluorescence were used to assess the spatio-temporal expression of peroxisome proliferator-activated receptor-γ coactivator-1α (PGC-1α), while western blot was adopted to detect the effect of TMP on PGC-1α. Neural apoptotic changes and neuronal survival were evaluated using the TUNEL method and Nissl staining, respectively. TMP treatment markedly increased PGC-1α expression, neuronal survival and BBB locomotor scores, while also reducing neural apoptosis. These results demonstrate that TMP is neuroprotective against contusive SCI, with the inhibition of neural apoptosis and increase of neuronal survival. The sustained expression of PGC-1α may partially contribute to the TMP-mediated neuroprotective effect.

## Introduction

Spinal cord injury (SCI), which is mostly caused by falls, sports injuries or traffic accidents, is a devastating injury that can lead to severe disability and lethal complications, including pulmonary system impairment [1, 2]. Data indicates that by the end of 2012, the prevalence of SCI across the globe reached a maximum of 1298 per million inhabitants [3]. Because of the large number of patients and severe complications of the disease, scientists have placed great effort into determining the underlying pathological mechanisms, in hope of developing more effective treatments. The complex course of SCI is mainly divided into the primary and the secondary injuries [4]. To date, it is widely accepted that the primary injury is an irreversible mechanical damage, while the secondary injury is reversible with some interventions. Thus, the major aim of SCI research is to develop therapeutic interventions, through the understanding of their mechanisms, to reduce and even reverse secondary spinal cord damage, and to regain motor functions.

Although the precise mechanism of the secondary pathogenic injury of SCI is still debatable, neuronal apoptosis has been implicated as the most damaging factor, which can lead to disastrous consequences [5, 6]. Recent promising evidence suggests an intimate link between peroxisome proliferator-activated receptor-γ coactivator-1α (PGC-1α) and neuronal survival in neurological disorders, such as Alzheimer’s disease, Parkinson’s disease, amyotrophic lateral sclerosis and cerebral ischemia [7–10]. PGC-1α is an important transcriptional coactivator closely linked to energy metabolism, and is involved in a variety of physiological and pathological processes. It is reported that, in certain neurological diseases, PGC-1α mainly plays a positive role by regulating mitochondria [11], which are also closely involved in regulating apoptosis [12, 13]. These studies have provided us with good inspiration for the development of SCI treatments. Although PGC-1α expression has been previously reported, its expression in spinal cord and alteration in SCI remain unclear. Further investigation is also required to determine if modulating its expression through pharmacological or other treatments has a positive impact on SCI.

For around a decade, our group has been researching SCI and traditional Chinese medicine therapy. Tetramethylpyrazine (TMP), which is of our particular interest, is an important chemical constituent that is extracted from the medicinal plant *Ligusticum wallichii*. It has been previously reported that TMP has a protective effect on the nervous system [14–16], but the mechanisms are poorly understood. Thus, the present study aimed to investigate the effect and underlying mechanism of TMP on neuronal protection closely related to PGC-1α activation in a clinically relevant model of SCI.

## Materials and Methods

### Animals and Experimental Design

Adult male Sprague–Dawley rats, weighing 220–250 g, were obtained from the Center of Experimental Animals, Central South University. All surgical interventions, perioperative care and drug administrations were performed in accordance with internationally accepted principles, and were approved by the Animal Use and Ethics Committee of Central South University. The rats were housed three (two after SCI modeling) per laboratory cage, with free access to food and water, in identical environments (temperature 22–24 °C; humidity 60–80 %). The experimental rats were randomly divided into the following three groups: Sham group, normal saline (NS) group and TMP group. Each group was divided into 6 subgroups (*n* = 10 per subgroup) according to the time of sacrifice: 1, 3, 7, 14, 21 and 28 days post-injury (dpi).

### Contusive Spinal Cord Injury Modeling

The establishment of contusive SCI model was performed as previously described [17], with slight modification. Briefly, the rats were intraperitoneally (i.p.) anesthetized with chloral hydrate, following which they received conventional skin preparation and antisepsis. Laminectomy was performed at thoracic vertebra level 10 (T10) in all groups, followed by contusive SCI of moderate severity (8 g weight × 3 cm vertical height free falling) in the NS and TMP groups. Successful signs of contusive SCI modeling were as follows: bleeding and edema in the T10 spinal cord, retracted flapping of both hind limbs, spasmodic sway of the tail, and flaccid paralysis after palinesthesia.

### Drug Administration

TMP was purchased from Tianjin Jinyao Amino Acid Co. Ltd., China. There were some differences in the effective dosage of TMP among other published reports [14, 18]. Taking the therapeutic and side effects into consideration, we ultimately chose 80 mg/kg as the application dosage in this study. TMP was injected i.p. daily for 5 consecutive days from day 3 post-injury in the TMP group, which was replaced by equal volumes of normal saline in the NS group.

### Behavioral Assessment

Behavioral assessments were performed prior to surgery and at 1, 3, 7, 14, 21 and 28 days post-injury in all three groups, based on a 21-point Basso–Beattie–Bresnahan (BBB) Locomotor Rating Scale, where 0 reflects no locomotion and 21 reflects normal motor functions [19]. Hind limb movements, trunk position and stability, tail position, stepping, coordination, paw placement and toe stretching were all observed over a 5 min period for each rat, by two independent examiners who were blinded to the experimental design.

### Tissue Collection

Spinal cord tissues were mainly harvested by two methods. The first method involved direct separation of about 1 cm length of spinal cord tissue at T10, after the rats were euthanized. The removed tissues were then immediately preserved in liquid nitrogen. These tissues were used for total RNA and protein extraction. The second method was as follows: the rats were deeply anesthetized and perfused with 300 ml 37 °C heparinized normal saline, through the ascending aorta, followed by 4 % paraformaldehyde (PFA) in 0.1 M PBS; the spinal cord tissues around T10 were then carefully removed, post-fixed in 4 % PFA overnight at 4 °C; serially dehydrated in 15 and 30 % sucrose solution until sinking, the dehydrated tissues were thereafter embedded in optimal cutting temperature (O.C.T.) compound, and frozen at −20 °C. The frozen O.C.T.-embedded tissues were sectioned at a 10-μm thickness for immunofluorescence, TUNEL and Nissl assays.

### Quantitative Real-Time PCR Analysis

Total RNA was extracted using the Trizol method, according to the manufacturer’s instructions (Invitrogen, USA). After reverse transcription was performed, quantitative real-time PCR (qRT-PCR) was analyzed in a BIO-RAD CFX96 Real-Time System, using SYBR^®^ Premix Ex Taq™ II (Takara, Japan). The primers were designed using NCBI primer-blast and synthesized by Sangon biotechnology (Shanghai, China) as follows: PGC-1α (forward: GGA CAC GAG GAA AGG AAG ACT A; reverse: GTA GCA CTG GCT TGA ATC TGT G) and β-actin (forward: CCC ATC TAT GAG GGT TAC GC; reverse: TTT AAT GTC ACG CAC GAT TTC). The specificity of PCR products was guaranteed by melting curve analysis. The expression of PGC-1α was normalized to β-actin, and the gene expression was comparatively analyzed using the 2^−ΔΔCt^ method.

### Western Blot Analysis

Total proteins were extracted using RIPA buffer and protease inhibitor phenylmethanesulfonyl fluoride (Beyotime, China). Protein concentrations were determined using the BCA method. Then, equal amounts of protein extracts were separated in an 8 % gel by SDS-PAGE, followed by transfer onto a polyvinylidene fluoride (PVDF) membrane. Blocking was performed for 1 h with 5 % skim milk in 0.05 % Tween-20 in PBS (PBST) at room temperature. Thereafter, the PVDF membrane was incubated with the primary antibody in 5 % IgG-free BSA (Genview, USA) overnight at 4 °C, followed by incubation with the horseradish peroxidase-conjugated secondary antibody for 1 h, after rinsing in PBST. Then, the membrane was rinsed in washing buffer and the signal was detected with enhanced chemiluminescence. Primary antibodies included PGC-1α (Novus, USA; 1:1500) and β-actin (BBI, China; 1:1000). Band intensities were quantified using Image J software. Values were normalized to β-actin in all samples.

### Immunofluorescence Assay

Immunofluorescence staining was performed on frozen sections. In brief, sections were rinsed in PBS and treated with 0.3 % Triton X-100 in PBS to disrupt the membranes. After blocking with 10 % normal goat serum for 1 h to reduce nonspecific staining, sections were incubated with the primary antibodies, mouse anti-beta III tubulin (Abcam, USA, 1:100) and rabbit anti-PGC-1α (Novus, USA, 1:150) overnight at 4 °C for 24 h. After rinsing in PBS, the secondary antibodies, Alexa Fluor 488 goat anti-mouse IgG (H + L) (Jackson, USA, 1:600) and Cy3 goat anti-rabbit IgG (H + L) (Jackson, USA, 1:800), were added onto sections in 5 % blocking solution, to incubate at room temperature for 30 min. Then, the sections were counterstained with DAPI (Sigma, USA), and imaged using a Leica DFC310 FX microscope (Leica, Japan).

### TUNEL Assay

Terminal deoxynucleotidyl transferase-mediated dUTP nick end labeling (TUNEL) staining was performed on frozen sections, to detect apoptotic changes of neural cells after contusive SCI, using the TUNEL staining apoptosis detecting kit (Nanjing KeyGen Biotech Co. Ltd., China) according to the manufacturer’s protocol. To reveal total cells, we counterstained the same sections with hematoxylin. The number of TUNEL-positive and total neural cells was counted in five different microscopic fields per section, and the TUNEL-positive percentage was calculated.

### Nissl Assay

Nissl staining was performed on frozen sections 5 mm rostrally to the lesion epicenter, with Nissl solution (0.1 % Cresyl violet) for 20 min. After rinsing in distilled water, the sections were differentiated in 95 % ethyl alcohol. Then, the sections were rinsed in 70 % ethyl alcohol, dehydrated in increasing concentrations of ethyl alcohol, and cleared in xylene. The cells containing Nissl bodies were considered as neurons, and those with typical neuronal morphology were counted per section. One out of every two sections was taken and a total of five sections were counted for each group to determine neuronal survival. A typical neuron exhibited a large amount of granule-like dense bluish violet staining indicating Nissl bodies in a regularly polygonal cell body. In a damaged neuron, Nissl bodies decreased in number and density, and the cell body became relatively small and irregular.

### Statistical Analysis

Statistical analysis was performed using SPSS 19.0 software (IBM, USA). Data were presented as the mean ± standard deviation (SD). The statistical significance of differences was determined using one-way analysis of variance (ANOVA) followed by Bonferroni post hoc test for multiple comparisons, or Student’s unpaired t test between two groups. All analyses were based on at least three different experiments with duplicate samples. A value of *P* < 0.05 was considered to be statistically significant.

## Results

### Effect of TMP on Locomotor Function Recovery

We first examined the effect of TMP on the recovery of locomotor functions. To obtain a holistic view, BBB Locomotor Rating Scale was implemented in a relatively systematic observation period. In our study, BBB scores were performed both prior to injury and at 1, 3, 7, 14, 21 and 28 days post-injury.

**Fig. 1:**
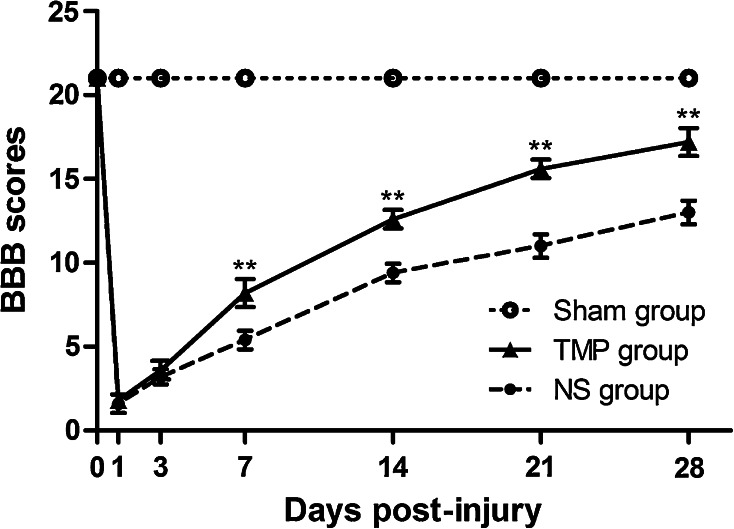
Effect of tetramethylpyrazine (TMP) on locomotor functional recovery. Prior to spinal cord injury (SCI), the Basso–Beattie–Bresnahan (BBB) scores of rats in both the TMP (n = 5) and normal saline (NS) (n = 5) groups were 21 points, which is the same score the sham group remains at for all time points. After contusive SCI, the BBB scores of rats in the NS and TMP groups rapidly decreased and then gradually increased. From 7 to 28 days post-injury, the BBB scores in the TMP group were significantly higher than the NS group. The fastest locomotor functional restoration occurred from 3 to 7 days post-injury in the TMP group. **P < 0.01 comparisons between the TMP and NS groups

As shown in Fig. 1, locomotor functions of rats exhibited a 21-point non-injured score in the Sham group. After contusive SCI, BBB locomotor rating scale was promptly decreased. At 1 day post-injury, BBB scores exhibited around two points. As the healing process progressed, a gradual recovery of locomotor functions could be found in both the NS and TMP groups, but the scores were significantly higher in TMP group at 7–28 days post-injury. TMP treatment markedly increased the recovery rate of locomotor function in rats with contusive SCI. Importantly, the fastest functional recovery occurred between days 3 and 7 post-injury in the TMP group. This showed that the administration of TMP was effective within this period, and correlated with the locomotor functional recovery at later time periods.

### Effect of TMP on PGC-1α Expression

Firstly, we investigated PGC-1α expression in physiological (as control) and injured spinal cord tissues, without any intervening measures. Following this, we examined whether any changes in expression occurred following TMP administration in a contusive SCI model, to better illustrate the intrinsic mechanism of the drug. We used qRT-PCR to assess PGC-1α expression in the T10 spinal cord at different time points. The spatial expression of PGC-1α was also examined by immunofluorescence staining.

**Fig. 2:**
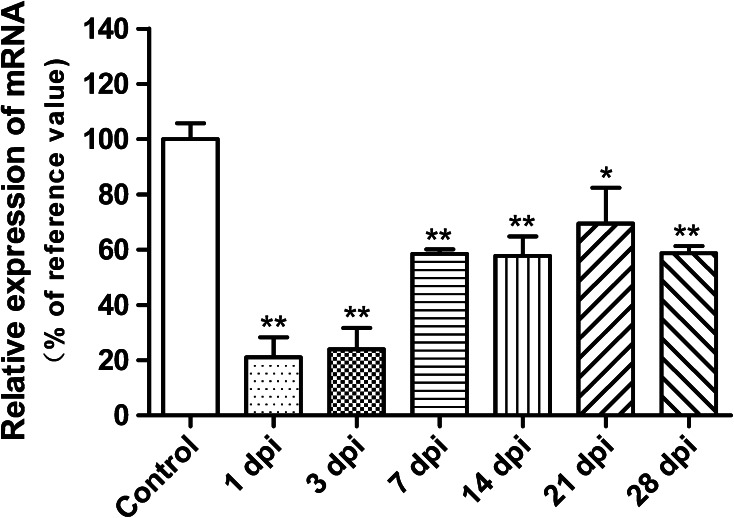
Quantitative RT-PCR analysis of the temporal expression of PGC-1α. It shows that after contusive spinal cord injury (SCI) (n = 3), PGC-1α expression in spinal cord significantly decreased compared with that of normal control (n = 3), followed by a gradual increase from 7 days post-injury (dpi). All expressions were normalized to β-actin. *P < 0.05, **P < 0.01 comparisons between each SCI group and the control group

**Fig. 3:**
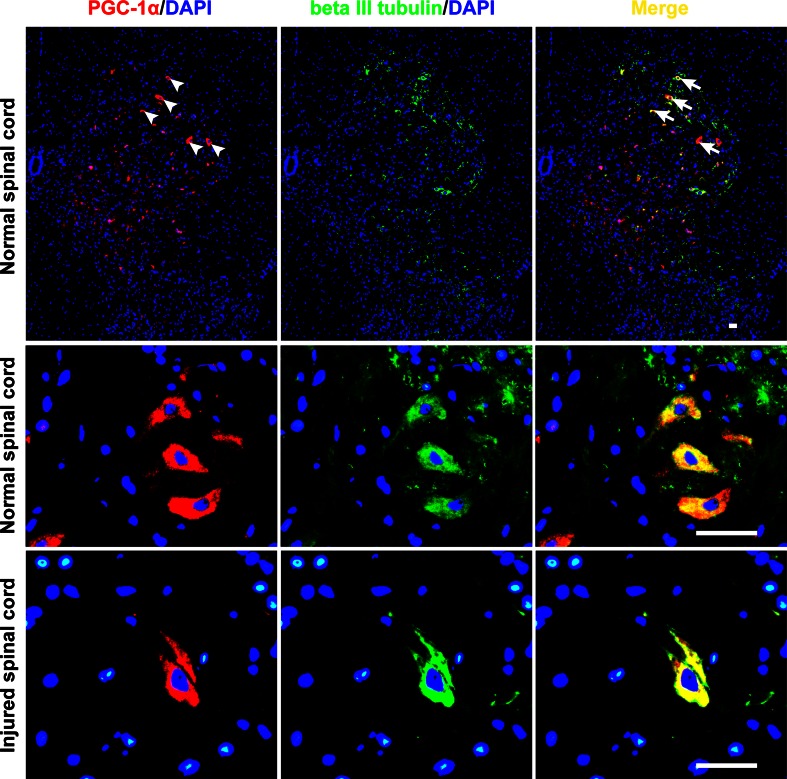
Immunofluorescence staining of the spatial expression of PGC-1α. The first row of images (**a**–**c**) demonstrates that PGC-1α is mainly expressed in the grey matter of the spinal cord in normal rats, and has a main co-localization with the neurons in the ventral horn. The β-III tubulin antibody was used to detect neurons.* White arrow heads* indicate the representative expression location of PGC-1α, and* white arrows* are representative of its co-localization with the neuronal marker. Double-staining at higher magnification in the second and third rows of images (**d**–**i**) clearly shows predominant overlap between neurons and PGC-1α in spinal cord tissues both pre- and post-contusive spinal cord injury. *Scale bar* 20 μm

Overall, PGC-1α expression significantly decreased after contusive SCI, followed by a gradual increase, in correlation with the spontaneous repair, until it reached a plateau. Specifically, relative to the normal control, the PGC-1α expression at 1 day post-injury decreased by about 80 %. From 7 days post-injury, an obvious increase occurred, but the expression level was still less than 60 % of the normal control at the end of our observation (Fig. 2). PGC-1α expression was mainly located in the grey matter of normal spinal cord. Besides, there was a high degree of overlap between neurons and PGC-1α, particularly in the ventral horn, while double staining devoid of neuronal marker could also be found. After injury, the main co-localization of neurons and PGC-1α could still be discovered despite the morphological abnormalities of the preserved neurons (Fig. 3). It is highly possible that PGC-1α plays a role of neuronal origin in protecting spinal cord homeostasis.

**Fig. 4:**
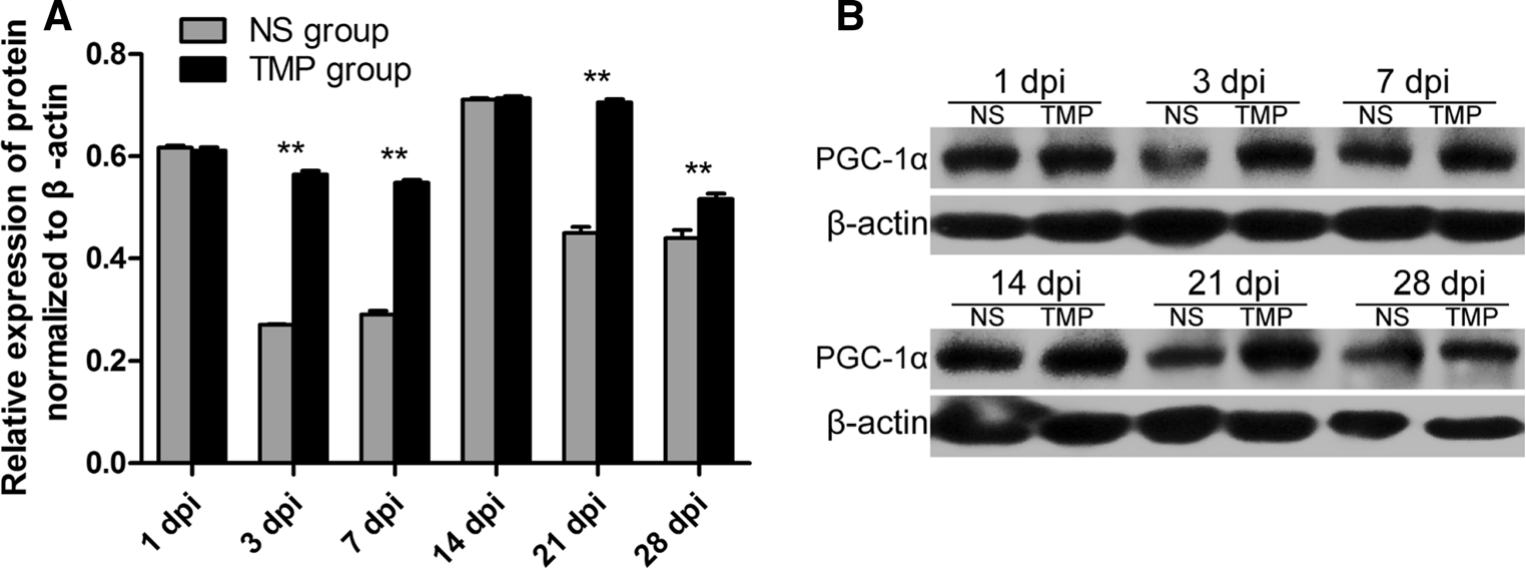
Effect of tetramethylpyrazine (TMP) treatment on PGC-1α expression. The quantitative data (**a)** and representative protein bands (**b**) of the western blot analysis of PGC-1α expression indicate that TMP treatment (n = 5) significantly increased the expression of PGC-1α at 3, 7, 21 and 28 days post-injury (dpi) compared with normal saline (NS) treatment (n = 5). **P < 0.01 comparisons between the TMP and NS groups

Intriguingly, PGC-1α expression was significantly higher in the TMP group than the NS group at 3, 7, 21, and 28 days post-injury (Fig. 4). This showed TMP treatment significantly upregulated PGC-1α expression in injured spinal cord tissues.

### Effect of TMP on Neural Apoptosis and Neuronal Survival

TMP significantly increased PGC-1α expression, especially at days 3 and 7 post-injury, and promoted fastest locomotor functional recovery; therefore, we believe that this is a crucial period for repairing the injured spinal cord with TMP treatment. We collected spinal cord tissues at 3 and 7 days post-injury to detect whether tissues in the TMP group exhibited less apoptosis and more survival.

**Fig. 5:**
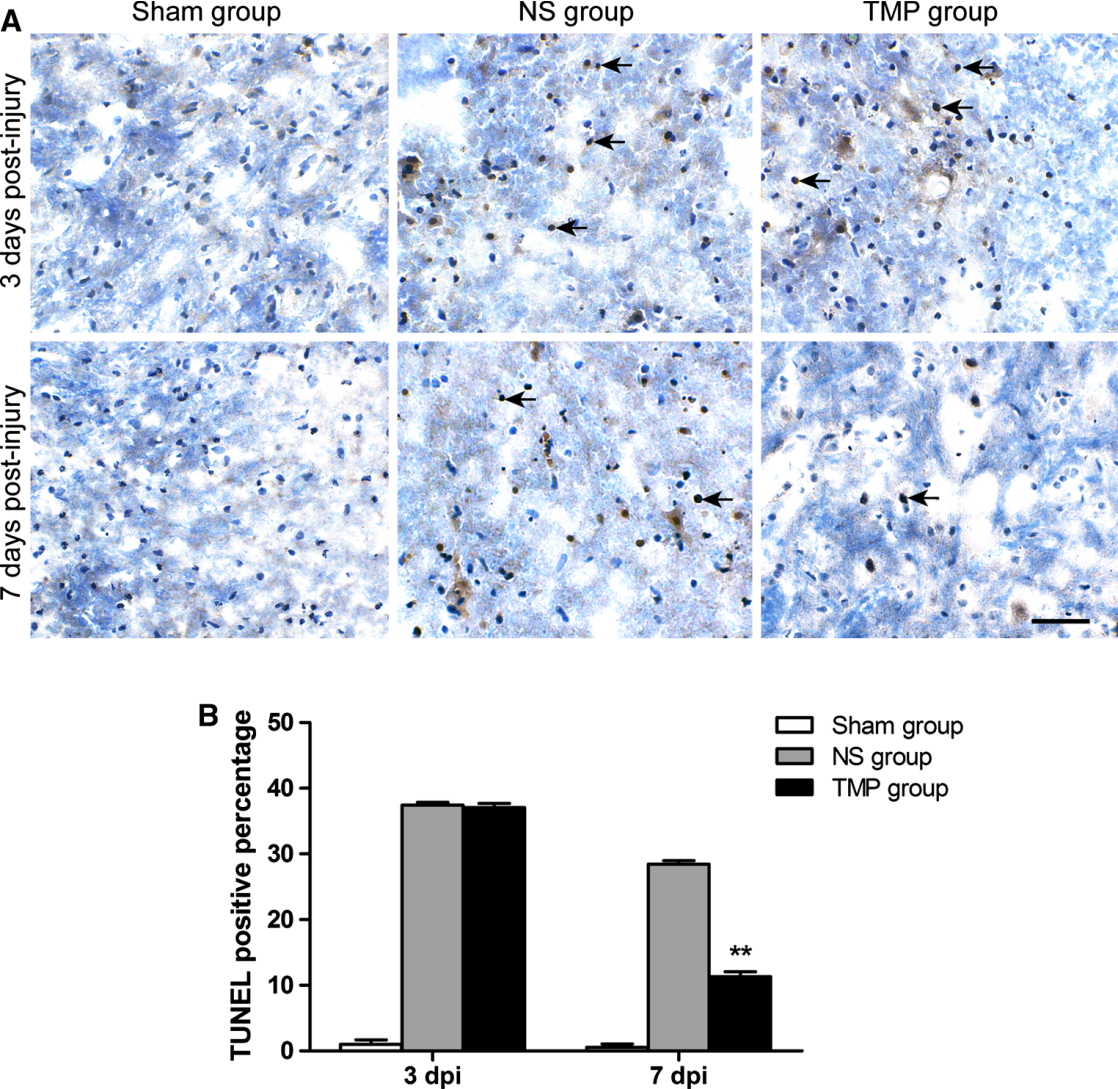
Effect of tetramethylpyrazine (TMP) treatment on neural apoptosis. Representative TUNEL staining (**a**) shows that apoptotic cells in T10 spinal cord were minimal in the sham group, but numerous in the normal saline (NS) and TMP groups. At 7 days post-injury (dpi), the apoptotic rate decreased in the TMP group compared with that of the NS group. Cell nuclei are stained blue.* Black arrows* indicate typical positive apoptotic staining.* Scale bar* 50 μm. The quantitative data (**b**) shows that the TUNEL-positive percentage in the TMP group (n = 5) was significantly lower than the NS group (n = 5) at 7 dpi. **P < 0.01 comparison between the TMP and NS group

The apoptotic change of neural cells was measured by TUNEL staining. In the Sham group, there was minimal apoptosis, so the TUNEL-positive percentage was extremely low. In contrast, the TUNEL-positive percentage was high in both the NS and TMP groups. However, TMP treatment significantly decreased the apoptosis rate of contusive SCI. At 7 days post-injury the TUNEL-positive percentage was approximately 28 and 11 %, respectively in the NS group and TMP group; the apoptosis rate of the TMP group decreased by about 60 % relative to the NS group (Fig. 5).

**Fig. 6:**
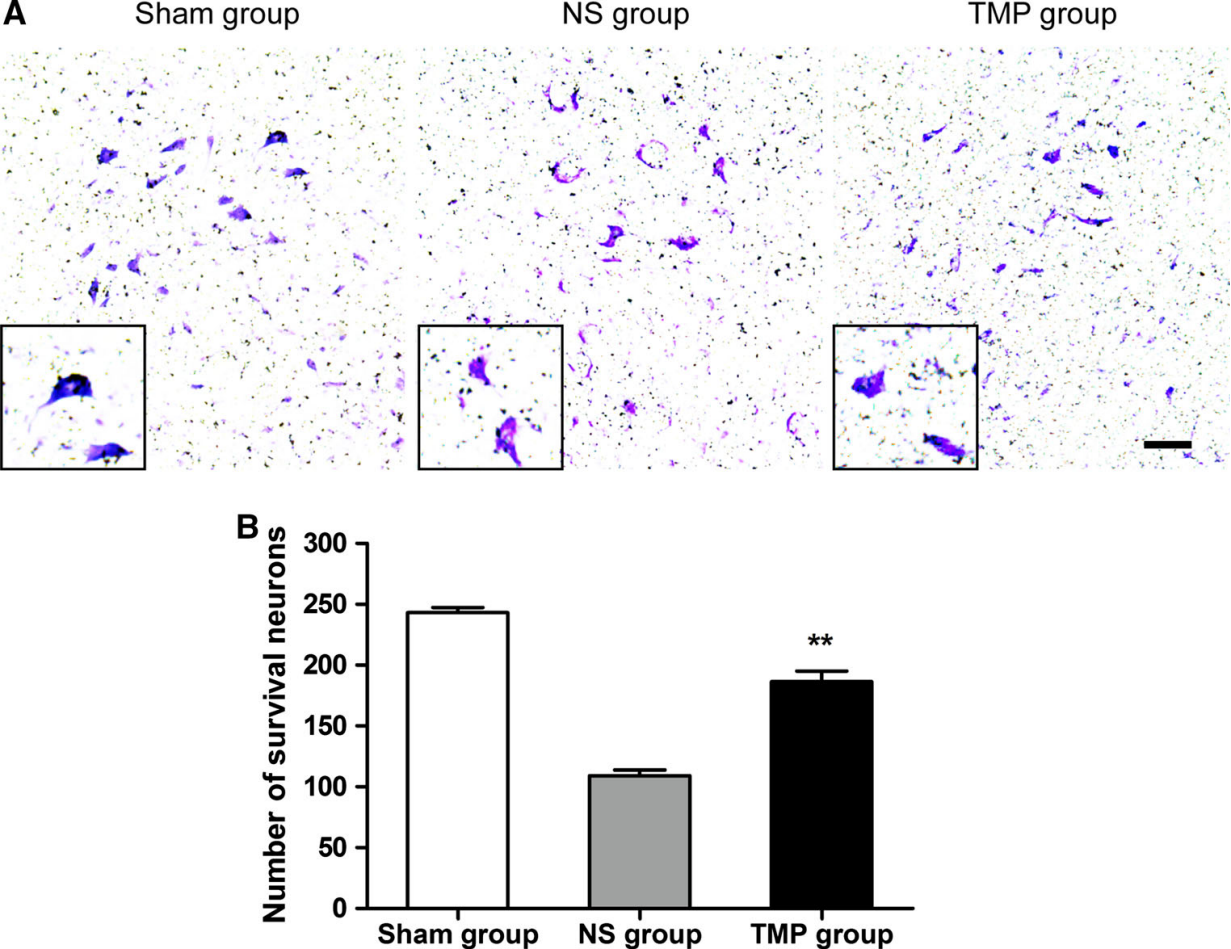
Effect of tetramethylpyrazine (TMP) treatment on neuronal survival. Representative Nissl staining (**a**) shows that at 7 days post-injury there were more surviving neurons in the TMP group than in the NS group, which were closer to the neurons in the Sham group. The mottled bluish violet stained typical morphological features of cells indicates neurons. Inside the black box are the examples of each group. *Scale bar* 100 μm. The quantitative data (**b**) demonstrates that the TMP group (n = 5) displayed a significantly increased number of neurons compared with that of the NS group (n = 5) at 7 days post-injury. **P < 0.01 comparison between the TMP and NS group

Next, we detected whether there was any difference in neuronal survival at 7 days post-injury using Nissl staining. Our results demonstrate that there were markedly more Nissl-positive cells with good morphology in the TMP group compared with that of the NS group (Fig. 6). This showed that TMP treatment promoted the survival of neurons after contusive SCI.

## Discussion

In the present study, we evaluated the neuroprotective effect of TMP on contusive SCI in a rat model. Our data showed for the first time that the expression of PGC-1α in spinal cord was decreased after contusive SCI. Following TMP treatment, there was a significant improvement in neurological recovery, as demonstrated by the BBB scores test, from day 3 post-injury. This neuroprotective effect was accompanied by a reduction of neural apoptosis, and increased PGC-1α expression and neuronal survival in TMP-treated animals. These findings indicate that the neuroprotective effect of TMP in contusive SCI may occur through the sustained expression of PGC-1α.

Clinically, the recovery of locomotor function is a key indicator of a better prognosis for SCI patients. It is also acknowledged that even a slight enhancement of locomotor function could bring considerable benefits to severely paralyzed patients. In the scientific field of SCI, the BBB Locomotor Rating Scale is the most widely accepted and commonly used open field test to evaluate motor function. Hence, we adopted this method in our study to assess the changes of motor function in rat models of contusive SCI, which is similar to the human condition [20]. Meanwhile, it has been suggested that integration of the CatWalk-based coordination method into the BBB Locomotor Rating Scale guarantees the validness of locomotor function assessment [21]. It is a reasonable suggestion, but the enormous value of BBB assessment by itself cannot be argued against. It has certain advantages, such as the exclusion of rats with incomplete damage and the revealing of a key period when maximum functional recovery occurred in our study, thus providing us with an affirmative scenario that the treatment of TMP on SCI is an efficient therapy.

TMP was initially used as a traditional medicine to improve blood circulation and remove blood stasis in the treatment of cerebral thrombosis, coronary heart disease, angiitis, and other disorders. Recently, it has been supported by several studies that TMP also plays a direct role in protecting neural tissue and cells [14, 15, 22]. Increasing emphasis is being placed on the application of TMP in neurovascular diseases. Nevertheless, because of the lack of publications on the molecular mechanism of TMP in the treatment of SCI, further investigation is still needed to elucidate the specific regulatory mechanism and lay the foundation for its extended clinical application. Following the mounting evidence on the role of PGC-1α in neurological diseases of the brain, we decided to investigate if PGC-1α expression changes after contusive SCI, and if TMP treatment also affects its expression. Comparing to normal spinal cord, PGC-1α expression after SCI significantly decreased and did not return to normal levels by the end of our observation period. These data are of particular importance, as it indicates that PGC-1α has a correlation with the physiological function of spinal cord.

Thus, we further investigated the impact of TMP treatment on PGC-1α expression following SCI. In fact, the gene of PGC-1α has multiple regulatory points and its transcriptional co-activation can be manipulated in combination with protein modification [23]. It is also well-known that the protein is the function executor of a specific gene, so western blot was the mainly adopted method to assess the effect of TMP on PGC-1α expression. Our results demonstrate that TMP can sustain the expression of PGC-1α in injured spinal cord. Moreover, PGC-1α is more likely to originate from neurons in the spinal cord, as we find that it mainly located in neurons in both normal and injured tissues. Meanwhile, the possibility of other origin such as glial cells cannot be excluded, since PGC-1α expression location does not totally overlap with neurons. Our results are similar to a study of amyotrophic lateral sclerosis, in which it is also speculated that the protective effects of PGC-1α are of neuronal rather than glial origin [9]. Furthermore, the TUNEL assay and Nissl staining results support the hypothesis that TMP treatment may partially regulate the PGC-1α expression at the protein level to protect against SCI.

To date, the specific association between PGC-1α expression and contusive SCI has not been elucidated. PGC-1α is a critical transcriptional co-activator enriched in mitochondria-rich tissues, and serves as an important regulator of cellular energy metabolism [24, 25]. However, the cellular effects of PGC-1α require the targeting of specific transcription factors, such as estrogen-related receptor-α (ERR-α), which has strong synergistic effects on PGC-1α activity [26, 27]. Nuclear respiratory factors (NRF-1/-2) are considered to be classical factors controlling the mitochondrial regulation of PGC-1α activity; however, ERR-α is believed to also influence mitochondrial gene expression and function in parallel to NRF-1/-2 dependent pathways [28]. By interacting with ERR-α, PGC-1α may have a direct effect on mitochondria in neurons following SCI. Another possible mechanism is that vascular endothelial growth factor (VEGF) mediates the final neuroprotection. ERR-binding sites have been identified within the VEGF promoter, and VEGF is perceived as a direct transcriptional target of the PGC-1α/ERR-α pathway [29, 30]. It is well known that VEGF is a critical factor in stimulating angiogenesis, but growing evidence suggests that the increased VEGF can directly induce neurotrophic and neuroprotective effects, which may be partially realized through binding to its receptor Flk-1 [31–33].

The treatment of SCI has never been an easy task. The central nervous system (CNS) is very complex, with neural and vascular components that are closely linked to each other, despite the existence of blood–brain barrier or blood-spinal cord barrier. Recently, the concept of the neurovascular unit (NVU) has drawn increasing attention to the comprehensive influence of neural and vascular factors on CNS diseases. The NVU, which consists of cellular and non-cellular compositions, including neurons, glia, microvessels, pericytes and the extracellular matrix, was initially been suggested to enhance the overall understanding of stroke [34, 35]. Now, more and more emphasis has been put on it when dealing with CNS diseases. This change in perception of the NVU is largely a result of investigations targeting the individual involvement of blood vessels or nerve factors in CNS repair not harvesting significant therapeutic effects. It is believed that the NVU is also present in the spinal cord, although morphological and functional differences exist between the blood–brain and blood-spinal cord barriers [36, 37]. Intriguingly, PGC-1α appears to be a factor that can exert a positive influence both on neural and vascular compositions. This speculation is supported by a recent in vitro study, where PGC-1α played a beneficial role in hypoxic preconditioning to both endothelial and neuronal cells [38]. In our future research, we will also focus on whether PGC-1α has a vascular protective effect. This is of particular importance, as if this gene has a unique capacity to exert multiple influences on neurovascular compositions of the spinal cord, the traditional Chinese medicine TMP could provide a powerful tool to efficaciously treat SCI.

In conclusion, our data implicate the spatial and temporal expression of PGC-1α in rat models of contusive SCI. We also highlight for the first time that the TMP-mediated induction of neuroprotective responses against SCI may partly involve sustaining PGC-1α expression. Although further research is needed to elucidate the entire signaling pathway, our present study provides strong support for the use of TMP treatment in SCI. These data may facilitate a better understanding of the benefits of TMP and warrant further study of this drug for the treatment of neurovascular diseases.
